# Supplementary material for: Human endogenous retroviruses form a reservoir of T cell targets in hematological cancers
Source: Nat Commun. 2020 Nov 9;11:5660. doi: 10.1038/s41467-020-19464-8 (PMC7653045; doi:10.1038/s41467-020-19464-8)
Supplement: Supplementary file 1 — Supplementary Information [file 41467_2020_19464_MOESM1_ESM.pdf]

Human endogenous retroviruses form a reservoir of T cell targets in hematological cancers

Sunil Kumar Saini[1*], Andreas Due Ørskov[2,3*], Anne-Mette Bjerregaard[1*], Ashwin Unnikrishnan[4,5], Staffan Holmberg-Thydén[1,6], Annie Borch[1], Kathrine Valentini Jensen[1], Govardhan Anande[4,5], Amalie Kai Bentzen[1], Andrea Marion Marquard[1], Tripti Tamhane[1], Marianne Bach Treppendahl[2], Anne Ortved Gang[6], Inge Høgh Dufva[6], Zoltan Szallasi[7,8], Nicola Ternette[9], Anders Gorm Pedersen[7], Aron Charles Eklund[7], John Pimanda[4,5,10], Kirsten Grønbæk[2,3,11], Sine Reker. Hadrup[1†]

[1]Department of Health Technology, Section of Experimental and Translational Immunology, Technical University of Denmark, Kongens Lyngby, Denmark.
[2]Department of Haematology, Rigshospitalet, Copenhagen University Hospital, Copenhagen, Denmark.
[3]Biotech Research and Innovation Centre (BRIC), University of Copenhagen, Copenhagen, Denmark.
[4]Adult Cancer Program, Lowy Cancer Research Centre, UNSW, Sydney, NSW 2052, Australia.
[5]Prince of Wales Clinical School, UNSW, Sydney, NSW 2052, Australia.
[6]Department of Haematology, Herlev Hospital, Copenhagen University Hospital, Herlev, Denmark.
[7]Department of Health Technology, Section of Bioinformatics, Technical University of Denmark, Kongens Lyngby, Denmark.
[8]Computational Health Informatics Program (CHIP), Boston Children's Hospital, Harvard Medical School, Boston, MA, United States.
[9]Nuffield Department of Medicine, University of Oxford, Oxford, United Kingdom.
[10]Haematology Department, South Eastern Area Laboratory Services, Prince of Wales Hospital, Randwick, NSW 2031, Australia.
[11]Novo Nordisk Foundation Center for Stem Cell Biology (DanStem), University of Copenhagen, Copenhagen, Denmark.
*These authors contributed equally to this work.
[†]Corresponding author. Email: sirha@dtu.dk

Supplementary Material

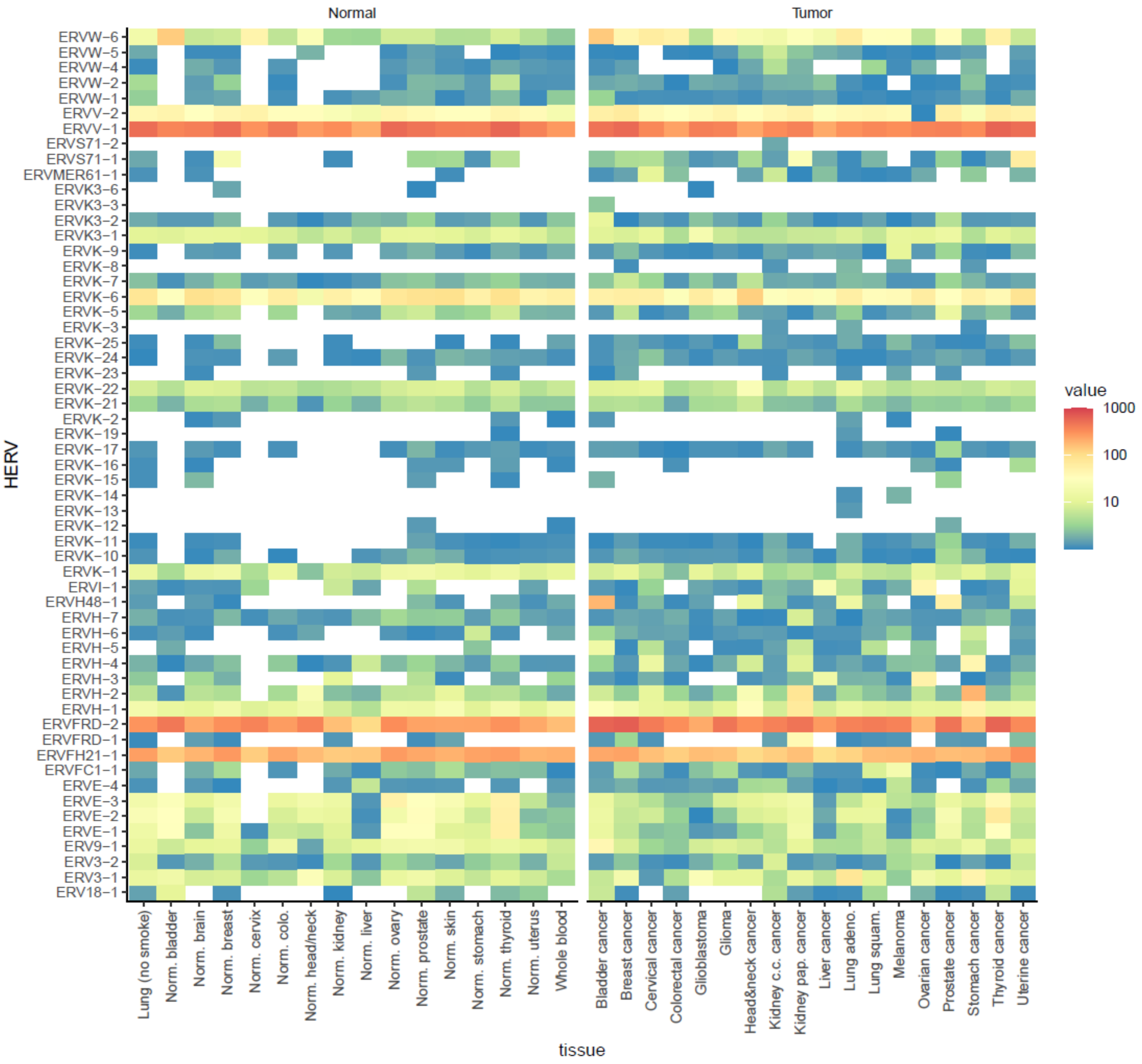

Supplementary Fig. 1. Expression of 66 HERVs, used for HERV-associated T cell antigen prediction, in normal and healthy tissues as reported by Rooney et al (ref. 12). Data plotted for 57 of the 66 HERVs showing expression at more than one read per million (RPM).

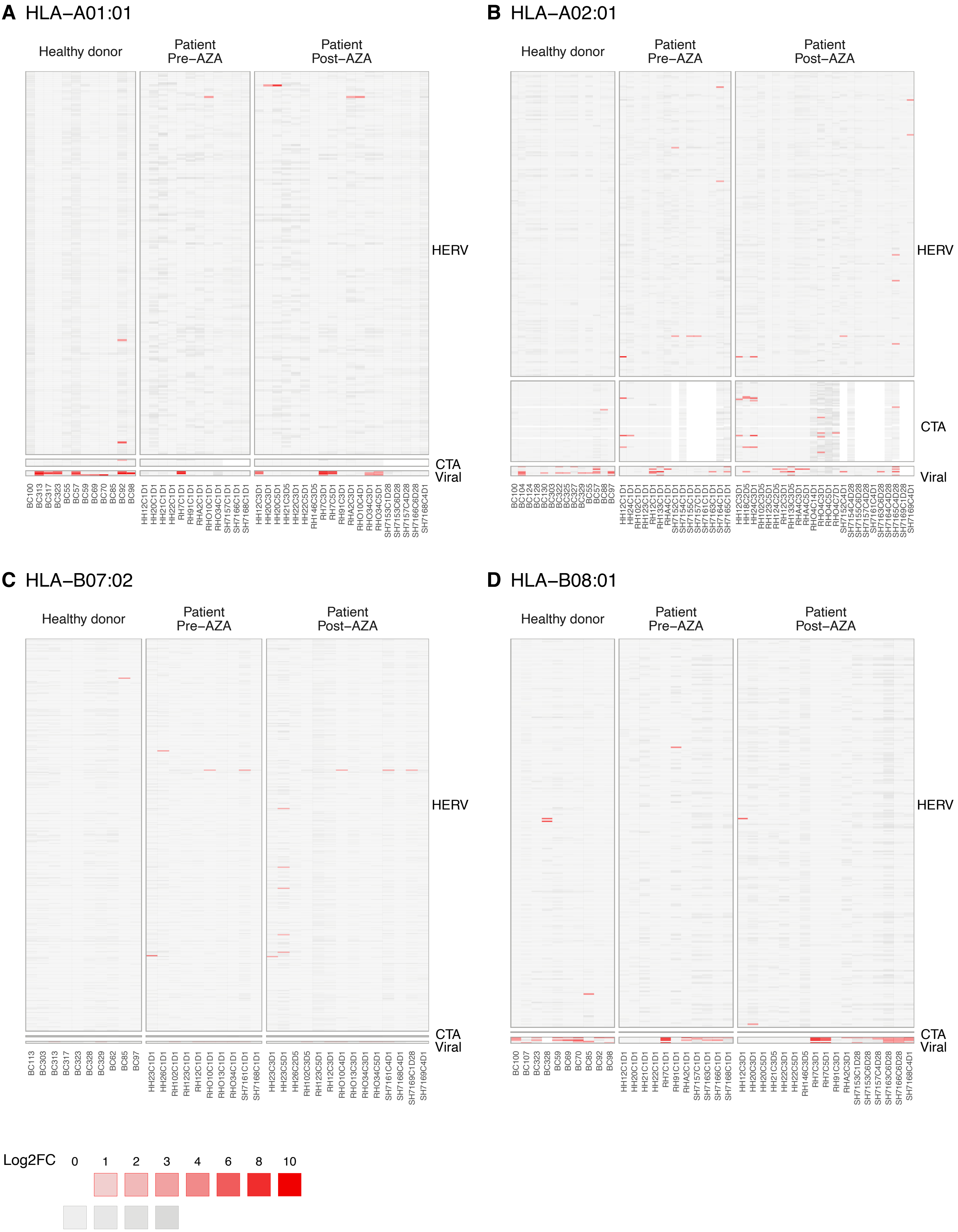
A HLA−A01:01
Healthy donor
Patient Pre−AZA
Patient Post−AZA
HERV
CTA
Viral
BC100 BC313 BC317 BC323 BC55 BC57 BC59 BC69 BC70 BC85 BC92 BC98
HH12C1D1 HH20C1D1 HH21C1D1 HH22C1D1 RH7C1D1 RH91C1D1 RHA2C1D1 RHO10C1D1 RHO34C1D1 SH7157C1D1 SH7166C1D1 SH7168C1D1
HH12C3D1 HH20C3D1 HH20C5D1 HH21C3D5 HH22C3D1 HH22C5D1 RH146C3D5 RH7C3D1 RH7C5D1 RH91C3D1 RHA2C3D1 RHO10C4D1 RHO34C3D1 RHO34C5D1 SH7153C1D28 SH7153C6D28 SH7157C4D28 SH7166C6D28 SH7168C4D1
B HLA−A02:01
Healthy donor
Patient Pre−AZA
Patient Post−AZA
HERV
CTA
Viral
C HLA−B07:02
Healthy donor
Patient Pre−AZA
Patient Post−AZA
HERV
CTA
Viral
BC113 BC303 BC313 BC317 BC323 BC328 BC329 BC62 BC85 BC97
D HLA−B08:01
Healthy donor
Patient Pre−AZA
Patient Post−AZA
HERV
CTA
Viral
BC100 BC107 BC323 BC328 BC59 BC69 BC70 BC85 BC92 BC98
Log2FC
0
1
2
3
4
6
8
10

Supplementary Fig. 2. Results of DNA-barcode based pMHC multimer analysis of HERV-, CTA-, and viral antigen libraries in patients and healthy donors. Log-fold change compared to background readout of sequenced DNA barcodes representing T cell reactivity detected in patients and healthy donors. Data encompass a cohort of 34 patients (Danish and Australian patients) and 27 healthy donors screened for HLA-matching peptide libraries of HERV-, CTA-, and viral epitopes. Results are segregated according to the four tested HLAs with each row representing a tested peptide of the three different categories. The patient cohort is divided based on before and after AZA treatment. T cell responses are shown based on Log2FC enrichment of the pMHC associated barcode compared to the baseline of the complete pMHC library, colored in red scale if significant (FDR <0.1%) and grey scale if no significant enhancement was found. Empty white regions are due to peptides not being included for the specific HLA matching samples.

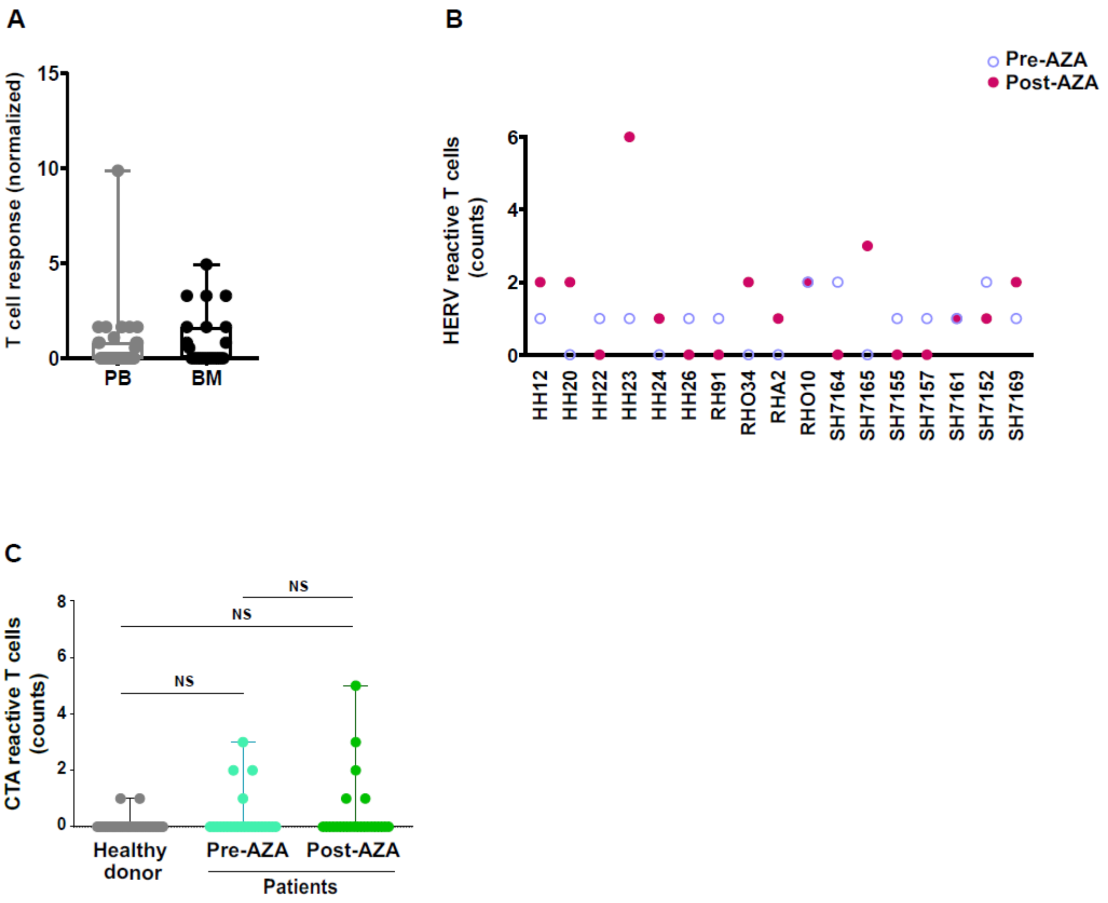

Supplementary Fig. 3. a HERV-specific T cell responses identified in peripheral blood and bone marrow. T cell responses shown in Figure 2 are here divided according to their sampling tissue; peripheral blood (PB) (Danish patients) or bone marrow (BM) (Australian patients). Data are shown as normalized (for the HLA analyzed) HERV-specific T cell responses detected out of the analyzed HERV library in each patient sample (combining T cell responses before and after AZA treatment), p=0.17 (Mann–Whitney–Wilcoxon test, two-tailed). Box plots showing the median, the lower and upper quartiles, and the whiskers as minimum and maximum values. PB, n=43; BM, n=24 (source data are provided as Source Data file). **b HERV-reactive T cells in pre- and post-AZA treatment samples.** Number of HERV-reactive CD8$^+$ T cell responses for each patient detected before and after AZA treatment. Data shown for 17 patients with one or more HERV-reactive T cells. **c CTA-reactive T cells identified using DNA-barcoded pMHC multimers (shown in Fig. 2a-d).** Comparison of CTA-reactive CD8$^+$ T cell responses identified in healthy donors and patients; p=0.24 (healthy donor vs pre-AZA), p=0.14 (healthy donor vs post-AZA) (Mann–Whitney–Wilcoxon test, two-tailed), and p=>0.99 (pre-AZA vs post-AZA, Wilcoxon Signed-Rank test, two-tailed). Box plots showing the median, the lower and upper quartiles, and the whiskers as minimum and maximum values. Healthy donors, n=27; pre-AZA, n=24; post-AZA, n=25 (source data are provided as Source Data file).

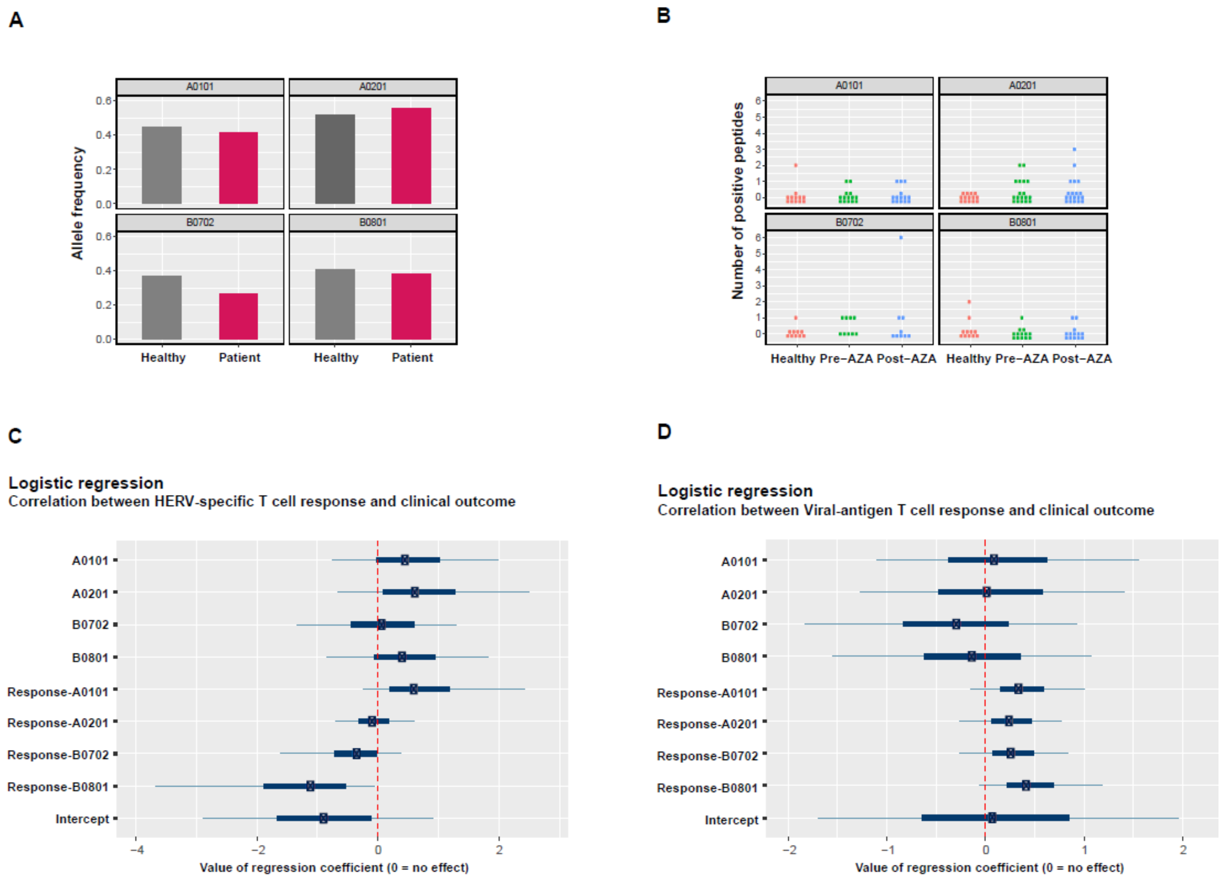

Supplementary Fig. 4. Distribution of the four HLAs and HERV-specific T cell responses segregated based on the HLA-restriction and logistic regression analysis correlating T cell responses with clinical outcome. a Distribution of the four HLA alleles analyzed for T cell recognition of HERV-derived peptides in patients and healthy donors. Data are shown as frequency of each HLA type in respective group. **b** HERV-reactive T cells identified in patients and healthy donors (shown in Fig. 2a-d) grouped according to their HLA restriction. **c** Logistic regression exploring the relationship between clinical outcome and T cell response to HERV peptides. The plot shows the posterior distribution of regression coefficients (posterior mean, 50% CI, and 90% CI). The following predictors were used: (1) The effect of the 4 HLA alleles in themselves: HLA-A*01:01, HLA-A*02:01, HLA-B*07:02, and HLA–B*08:01; (2) the number of peptides recognized for the indicated HLA allele, standardized to have zero mean and unit variance: response-A0101, response-A0201, response-B0702, response-B0801. Note that the credible intervals for most regression coefficients include 0, indicating no statistically significant effect of the explored predictors. The one exception is response-B0801 which appears to have a negative impact on the probability of being a responder. This is almost certainly statistical noise, and is caused by two non-responders that have a T cell response to HERV peptides for B8 (and no responders having a T cell response to these peptides). **d**

Logistic regression for predicting clinical outcome from the response to viral peptides. The plot shows the posterior distribution of regression coefficients (posterior mean, 50% CI, and 90% CI). Predictors are the four HLA alleles (as above), and the viral response to the viral peptides (count of recognized peptides, standardized as for HERV). All regression coefficients include zero in their 90% credible intervals indicating no statistically significant effect of the investigated predictors.

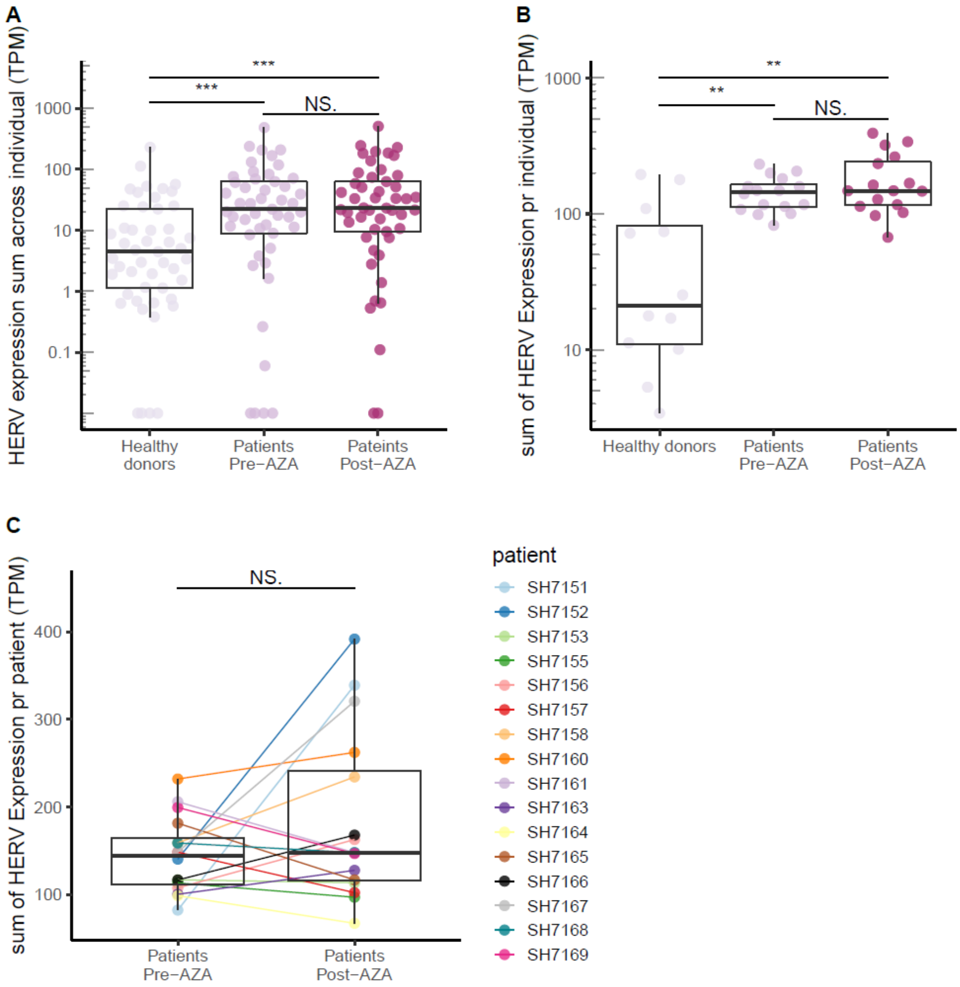

Supplementary Fig. 5. HERV expression analysis across patient and healthy donor samples. **a** The expression of each of the 49 HERVs across all individuals (healthy donors and patients before and after AZA treatment); p=6.5e-4, p=3.8e-4, and p=0.73, respectively). Healthy donors, pre-AZA, n=49, and post-AZA, n=49. **b** The sum of HERV expression of all 49 HERVs in each individual, split into groups of healthy donors and patients before and after AZA treatment; p=0.002, p=0.0015, and p=0.39, respectively). Healthy donors, n=12; pre-AZA, n=16; post-AZA, n=16. **c** Paired analysis of patients in B (p=0.40). For statistical tests a two-tailed Mann–Whitney–Wilcoxon test was used between healthy donors and patient groups, while a two-tailed Wilcoxon Signed-Rank test was used to compare patients before and after treatment. In boxplots the box shows the 1st quartile (Q1), the median, and the 3rd quartile (Q3), while whiskers extend to 1.5 times the interquartile range (IQR) on either side of the box (or to the minimum and maximum data values if these are less than 1.5 * IQR from Q1 and Q3).

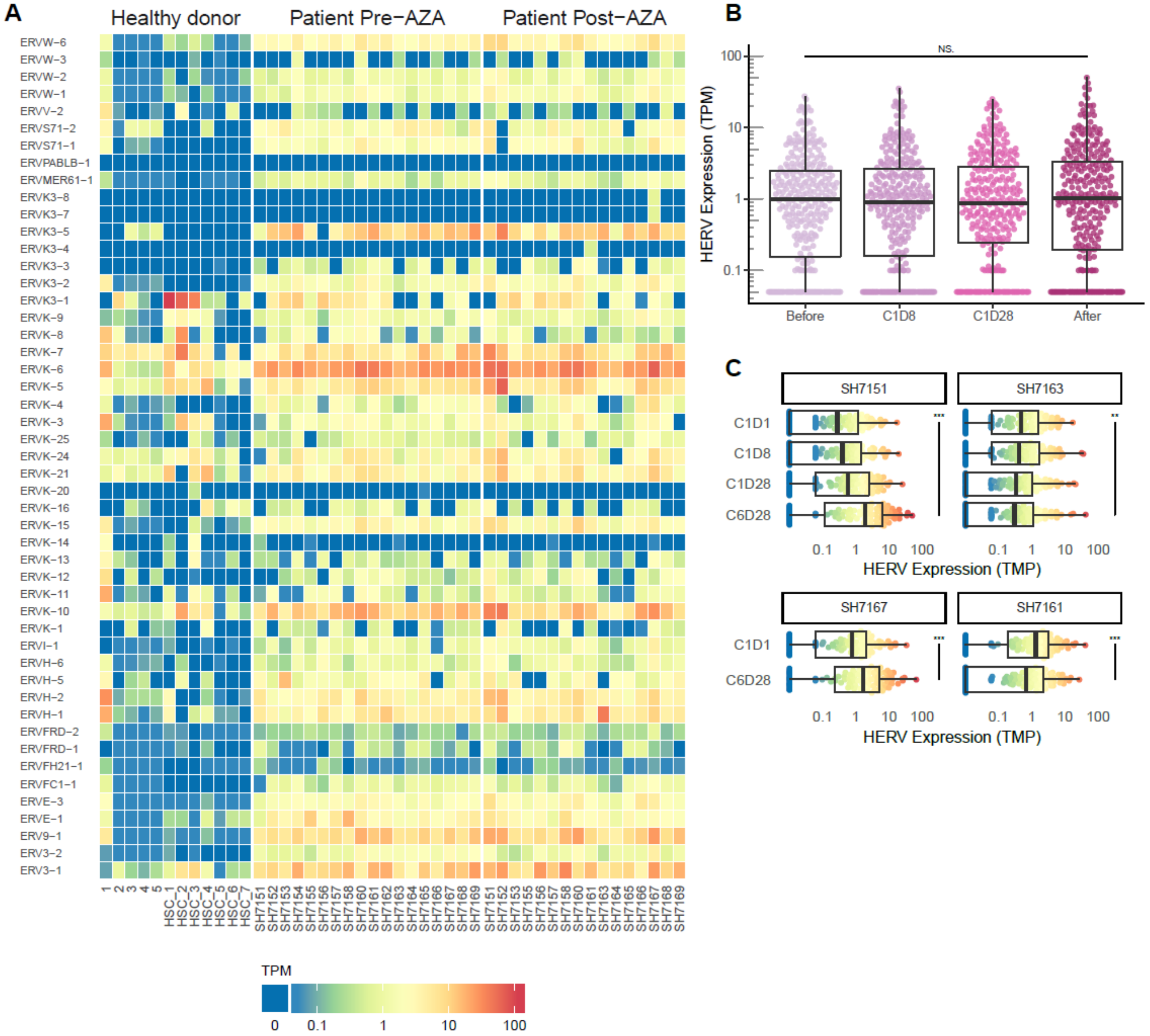

Supplementary Fig. 6. HERV expression analysis. a Heatmap showing HERV expression profiles of the 49 HERVs included in the T cell analysis of patients (Australian patients; n=18) before and after treatment (cycle 6 day 28) together with healthy donor samples (n=14). The expression is measured as transcripts per million reads (TPM) and all values below 0.05 TMP were set to 0.05 TPM, and above 150 TPM set to 150 TPM. **b** Expression profile of HERVs before, during, and after (C6D28) AZA treatment (mean of each HERV across the patient samples; p=0.24, Wilcoxon Signed-Rank test, two-tailed, n=294 for each group). **c** Individual examples comparing overall change in HERV expression pattern before and after treatment (SH7151, p=1.5e-13; SH7163, p=3.1e-3; SH7167, p=1.0e-13; SH7161, p=9.0e-8;

Wilcoxon Signed-Rank test, two-tailed). In boxplots the box shows the 1st quartile (Q1), the median, and the 3rd quartile (Q3), while whiskers extend to 1.5 times the interquartile range (IQR) on either side of the box (or to the minimum and maximum data values if these are less than 1.5 * IQR from Q1 and Q3).

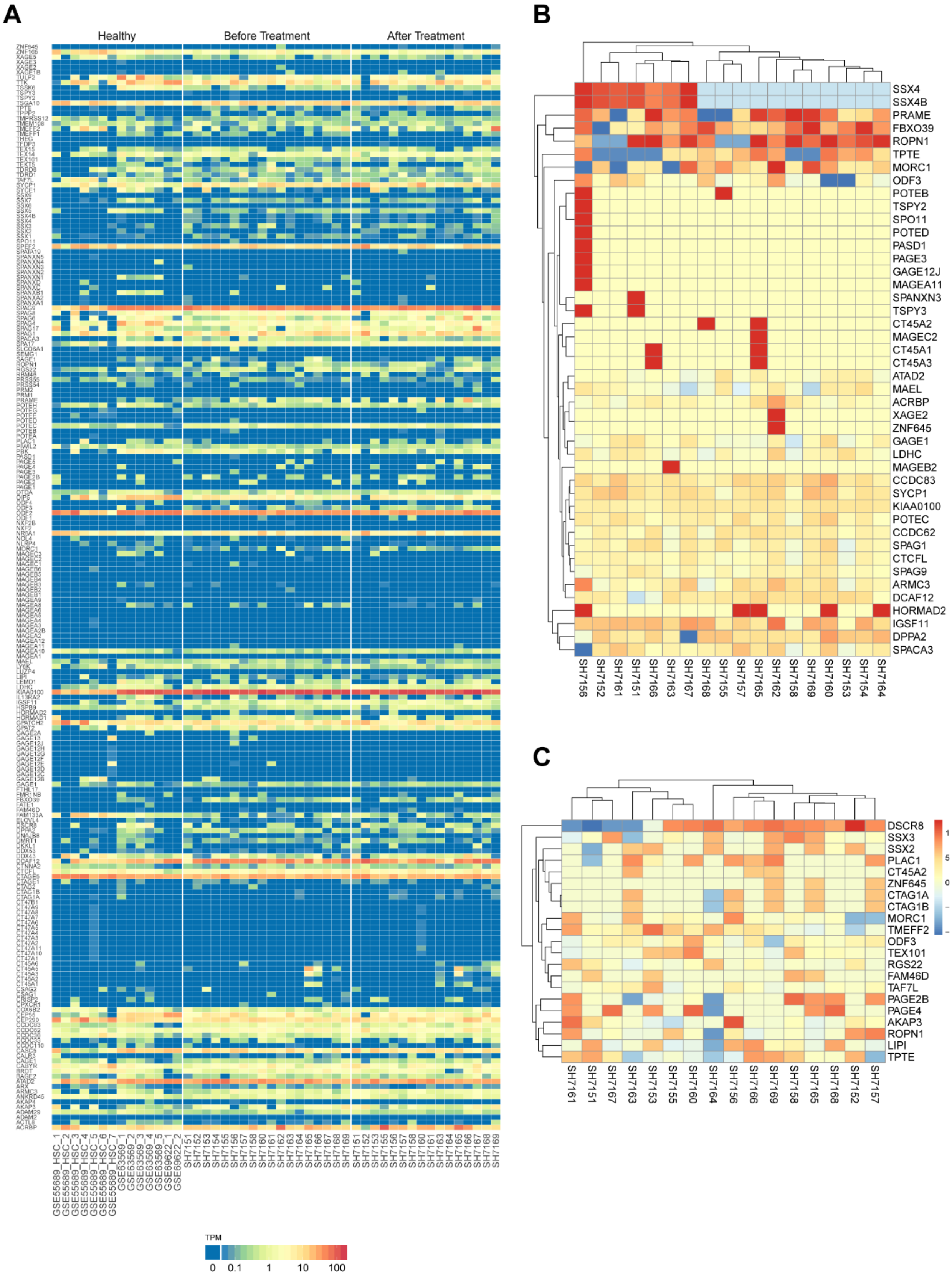
A
Healthy
Before Treatment
After Treatment
TPM
0 0.1 1 10 100
B
SSX4
SSX4B
PRAME
FBXO39
ROPN1
TPTE
MORC1
ODF3
POTEB
TSPY2
SPO11
POTED
PASD1
PAGE3
GAGE12J
MAGEA11
SPANXN3
TSPY3
CT45A2
MAGEC2
CT45A1
CT45A3
ATAD2
MAEL
ACRBP
XAGE2
ZNF645
GAGE1
LDHC
MAGEB2
CCDC83
SYCP1
KIAA0100
POTEC
CCDC62
SPAG1
CTCFL
SPAG9
ARMC3
DCAF12
HORMAD2
IGSF11
DPPA2
SPACA3
SH7156 SH7152 SH7161 SH7151 SH7166 SH7163 SH7167 SH7168 SH7155 SH7157 SH7165 SH7162 SH7158 SH7169 SH7160 SH7153 SH7154 SH7164
C
DSCR8
SSX3
SSX2
PLAC1
CT45A2
ZNF645
CTAG1A
CTAG1B
MORC1
TMEFF2
ODF3
TEX101
RGS22
FAM46D
TAF7L
PAGE2B
PAGE4
AKAP3
ROPN1
LIPI
TPTE
SH7161 SH7151 SH7167 SH7163 SH7153 SH7155 SH7160 SH7164 SH7156 SH7166 SH7169 SH7158 SH7165 SH7168 SH7152 SH7157

Supplementary Fig. 7. CTA expression analysis. a Heatmap (TPM, transcripts per million reads) of CTA genes expression analyzed from RNA-seq data of Australian patients (n=18) compared with healthy donors (n=14). Patient data are compared before and after treatment. Genes with expression above 150 TPM is set to 150 to avoid skewing. **b** Fold change for CTA genes showing increased expression of some CTAs in patients (before treatment) compared to healthy donors. Log fold change values above 5 is set to 5 and below -5 is set to -5. Only genes with a mean log fold change above 0 across all samples are illustrated. **c** Similar to B, comparing patient samples before and after treatment. CTA genes included in the analysis are selected from CTdatabase. The selected genes shown are according to a mean log fold change above 0 across all samples.

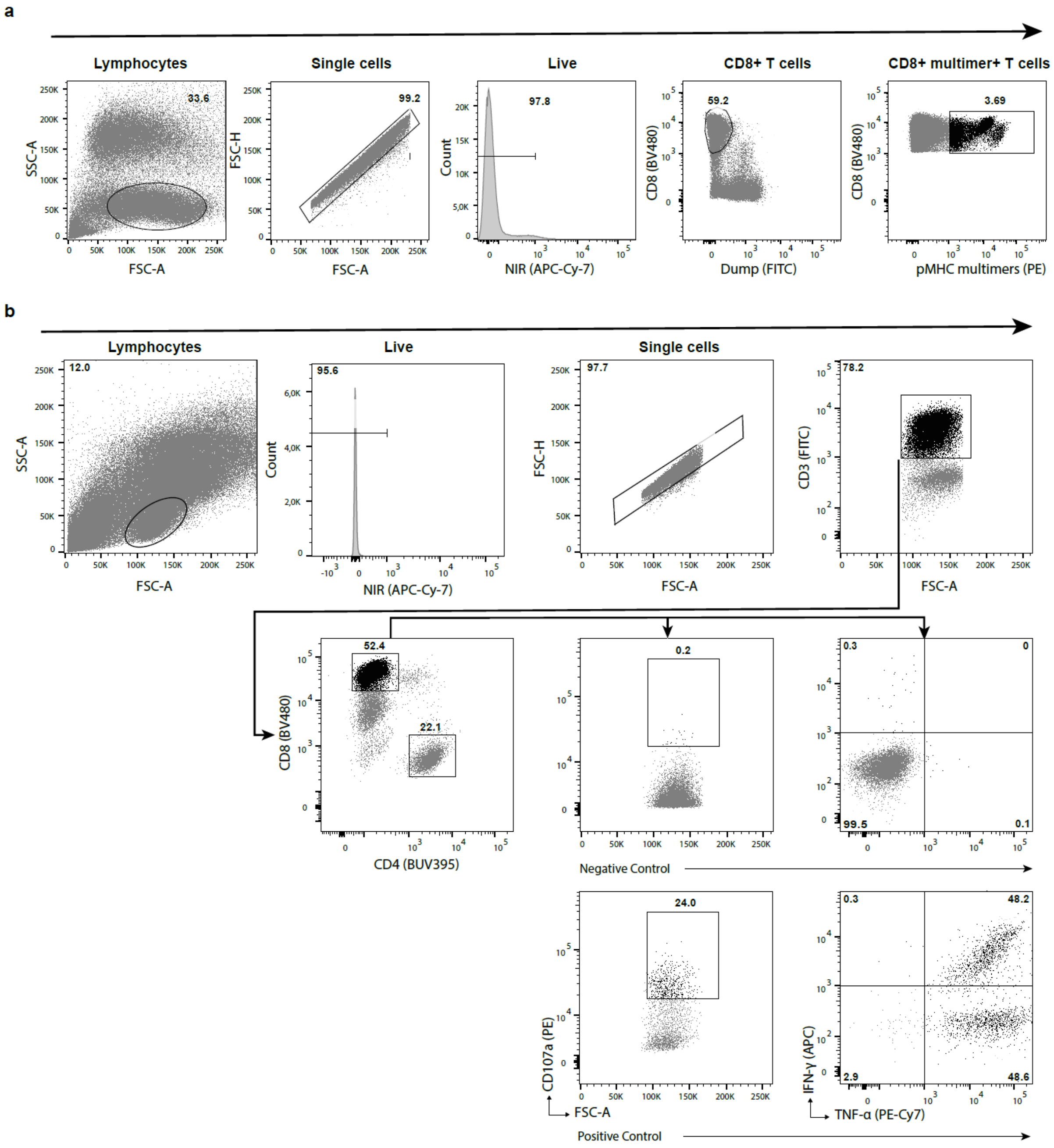

Supplementary Fig. 8. Gating strategies for flow cytometry analysis. a Gating used to sort DNA-barcoded pMHC-multimer (PE) positive CD8^{+} T cells specific to HERV, CTA and viral antigens in patient and healthy donor samples. Data shown in Fig. 2a-d are derived from multimer positive cells sorted using this strategy. **b** Flow cytometry gating for T cell functional analysis shown in Fig. 4a-c. Plots of negative and positive controls are shown here.

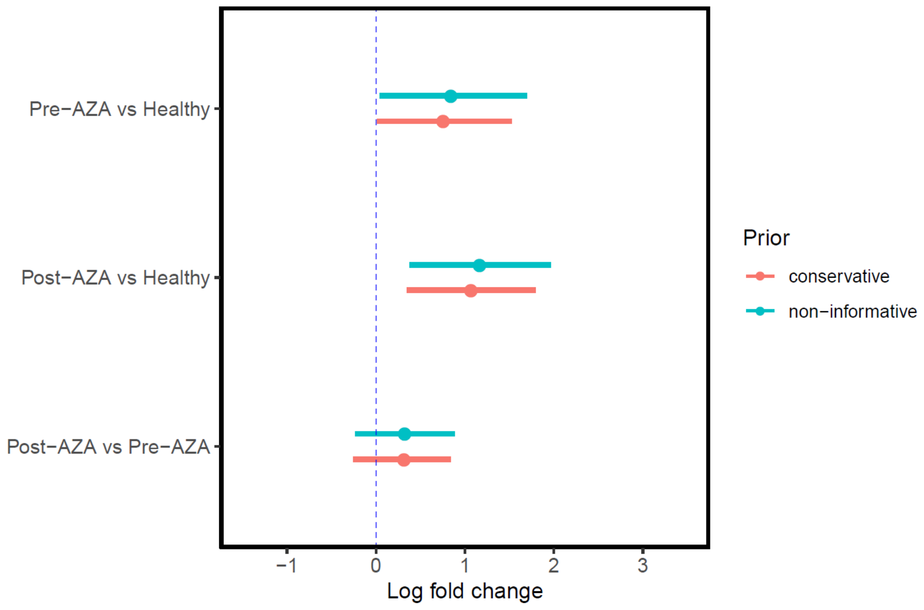

Supplementary Fig. 9. Sensitivity analysis of log fold change in proportion in HERV-reactive T cell recognition recognized using different priors. Comparison of the effects of two different priors on the estimates of the log fold change in proportion of HERV peptides recognized by T cells between pairs of cohorts (shown in Fig. 3c). The plot shows the posterior distribution of regression coefficients (posterior mean and 50% CI). Red distributions were estimated using a conservative prior (i.e., one that tries to keep estimates close to zero, unless the data support an effect); this is the prior used in the plots shown in the main text (normal (-3, 4) for intercept and normal (0, 1) for group effects). Green distributions show the same estimates using a non-informative prior (i.e., one that provides almost no information about the parameter estimates, thus giving more influence to the data; normal (0, 10) for intercept and for group effects). It can be seen that the conservative prior pulls the estimate slightly towards zero, but overall the estimates are almost identical.

Supplementary Table **1. Characteristics of the patients included in the T cell screening.**

Patient ID	Disease - WHO	Sex	Age at Aza-start	Cytogenetics - IPSS	IPSS-R or CPSS	Response
RH-7	MDS/RAEB-II	F	83	Intermediate	Very high	Y
RH-12	MDS/RAEB-II	M	55	Good	High	N
RH-91	AML	M	54	Intermediate	Very high	Y
RH-123	CMML-II	F	67	Normal	INT-2	N
RH-133	MDS/RAEB-II	M	57	Intermediate	High	N
RH-O4	CMML-II	F	65	Good	INT-1	Y
RH-O10	AML (>30%blasts)	F	72	Poor	(AML)	Y
RH-O13	AML (>30%blasts)	M	75	Poor	(AML)	N
RH-O34	CMML-II	M	64	Good	INT-2	Y
RH-A2	MDS/RAEB-II	M	77	Good	High	Y
RH-A4	MDS/RAEB-II	F	60	Very poor	High	Y
RH-102	MDS/RAEB-II	M	75	Intermediate	Very high	N
RH-124	MDS/RCMD	F	51	Poor	High	Y
RH-146	CMML-II	M	74	Intermediate	High	N
HH-12	AML (>30% blast)	M	78	Normal	(AML)	N
HH-18	MDS/RARS	F	81	Poor	Intermediate	n/a
HH-20	MDS/RAEB-II	F	75	Poor	Very high	N
HH-21	AML	M	80	Poor	(AML)	N
HH-22	MDS/RCMD	M	62	Good	Low	N
HH-23	MDS/RARS	F	58	Good	Low	N
HH-24	MDS/RCUD	F	73	Good	Low	N
HH-26	MDS/RCMD	M	12	Good	Low	N
SH-PD7152	CMML-II	F	65	Good	INT-2	N
SH-PD7153	CMML-II	F	70	Good	INT-1	Y
SH-PD7154	CMML-II	M	82	Very good	INT-2	Y
SH-PD7155	CMML-II	F	70	Poor	High	Y
SH-PD7157	CMML-II	M	59	Good	INT-2	Y
SH-PD7161	MDS/RAEB-II	F	75	Good	High	Y
SH-PD7163	MDS/RAEB-II	F	69	Very good	High	Y
SH-PD7164	MDS/RAEB-2	M	75	Good	Intermediate	Y
SH-PD7165	MDS/RAEB-2	M	73	Good	n/a	Y
SH-PD7166	MDS/RAEB-2	M	69	Intermediate	High	N
SH-PD7168	MDS/RAEB-2	F	77	Very poor	Very high	Y
SH-PD7169	MDS/RCMD	M	75	Very poor	High	N

Abbreviations:

- Disease at initiation of azacitidine treatment according to the 2008 revision of the World Health Organization (WHO) - myelodysplastic syndrome (MDS), refractory anemia with excess of blasts (RAEB), refractory anemia (RA), refractory cytopenia with multilineage dysplasia (RCMD), refractory anemia with ring sideroblasts (RARS), refractory cytopenia with unilineage dysplasia (RCUD), chronic myelomonocytic leukemia (CMML) and acute myeloid leukemia (AML).
- IPSS=International Prognostic Scoring System. At time of diagnosis.
- IPSS-R=Revised International Prognostic Scoring System. At time of diagnosis.
- CPSS=CMML-specific prognostic scoring system. At time of diagnosis.
- IPSS-R/CPSS prognosis: Score “Low”=best prognosis; Score “Very high”=poorest prognosis. INT-1/2=intermediate 1/2.
- Cytogenetics – IPSS: “Very good” corresponds to best prognosis and “very poor” corresponds to poorest prognosis.
- Response is determined as complete remission, marrow complete remission, partial remission and hematological improvement.
- Non-response as stable disease without, disease progression and treatment failure.

Supplementary methods

Bayesian model of the connection between clinical outcome and reactivity to HERV or viral antigens (Supplementary Fig. 4c,d)

To investigate the connection between clinical outcome (where patients are divided into responders or non-responders) and the immune reactivity towards HERV or viral peptides, we used a logistic regression model. Specifically, the aim was here to predict the probability that a patient will be a responder, using as predictors (1) the presence or absence of the 4 HLA alleles, (2) the number of antigen peptides recognized for each HLA allele (standardized to have zero mean and unit variance):

$$p(responder) = logistic(\beta_0 + \beta_{HLA} x_{HLA} + \beta_{peptide\ count} x_{peptide\ count}) \qquad (7)$$

Here β_0 is the intercept, corresponding to the probability of being a responder if all predictors are 0, χ_{HLA} is a length-4 vector of indicator variables that specify which of the 4 HLA alleles are present, β_{HLA} is the corresponding length-4 vector of regression coefficients, $\chi_{peptide\ count}$ is a length-4 vector of counts giving the number of peptides recognized by each HLA allele (standardized to have zero mean and unit variance), and $\beta_{peptide\ count}$ is the corresponding vector of regression coefficients. We used hierarchical priors on the regression coefficient vectors to regularize the estimates. Specifically, the 4 parameters in each coefficient vector were assumed to be drawn from a higher-level normal distribution which mean and variance were themselves estimated from the data. This has the effect of shrinking the individual estimates towards the overall mean, thus helping avoiding overfitting to the small data set used here.
