# Supplementary material for: Human endogenous retroviruses form a reservoir of T cell targets in hematological cancers
Source: Nat Commun. 2020 Nov 9;11:5660. doi: 10.1038/s41467-020-19464-8 (PMC7653045; doi:10.1038/s41467-020-19464-8)
Supplement: Supplementary file 3 — Reporting Summary [file 41467_2020_19464_MOESM3_ESM.pdf]

nature research

Corresponding author(s): Sine Reker Hadrup

Last updated by author(s): Sep 28, 2020

Reporting Summary

Nature Research wishes to improve the reproducibility of the work that we publish. This form provides structure for consistency and transparency in reporting. For further information on Nature Research policies, see Authors & Referees and the Editorial Policy Checklist .

Statistics

For all statistical analyses, confirm that the following items are present in the figure legend, table legend, main text, or Methods section.

n/a	Confirmed	
☐	☒	The exact sample size (*n*) for each experimental group/condition, given as a discrete number and unit of measurement
☒	☐	A statement on whether measurements were taken from distinct samples or whether the same sample was measured repeatedly
☐	☒	The statistical test(s) used AND whether they are one- or two-sided<br>*Only common tests should be described solely by name; describe more complex techniques in the Methods section.*
☒	☐	A description of all covariates tested
☐	☒	A description of any assumptions or corrections, such as tests of normality and adjustment for multiple comparisons
☐	☒	A full description of the statistical parameters including central tendency (e.g. means) or other basic estimates (e.g. regression coefficient) AND variation (e.g. standard deviation) or associated estimates of uncertainty (e.g. confidence intervals)
☐	☒	For null hypothesis testing, the test statistic (e.g. *F*, *t*, *r*) with confidence intervals, effect sizes, degrees of freedom and *P* value noted<br>*Give P values as exact values whenever suitable.*
☐	☒	For Bayesian analysis, information on the choice of priors and Markov chain Monte Carlo settings
☒	☐	For hierarchical and complex designs, identification of the appropriate level for tests and full reporting of outcomes
☒	☐	Estimates of effect sizes (e.g. Cohen's *d*, Pearson's *r*), indicating how they were calculated

Our web collection on statistics for biologists contains articles on many of the points above.

Software and code

Policy information about availability of computer code

Data collection	*Provide a description of all commercial, open source and custom code used to collect the data in this study, specifying the version used OR state that no software was used.*
Data analysis	Sequencing data from the DNA barcode-based screening was analyzed using Barracoda software version 1 (https://services.healthtech.dtu.dk/service.php?Barracoda-1.8)<br>All statistical tests were calculated in R analysis package (version 3.6.1), and using the software RStan (version 2.19.3) from within the R programming environment in RStudio (Carpenter et al., 2017; RStudio Team, 2018; R Core Team, 2019; Stan Development Team, 2020).<br>Preparation of data as well as post-processing and plotting of results was done using the “tidyverse” (version 1.3.0) and “tidybayes” (version 2.0.3) R-packages.<br>Flow cytometry data was analyzed in FACS DIVA software, version 8.02.<br>RNA-seq data were evaluated using the following programs: Trim Galore (version 0.4.0), FAstQC (version 0.11.2), cutadapt (version 1.9.1), and Kallisto (version 0.42.1).<br>Heatmaps were developed using pheatmap (version 1.0.12) in R analysis package.

For manuscripts utilizing custom algorithms or software that are central to the research but not yet described in published literature, software must be made available to editors/reviewers. We strongly encourage code deposition in a community repository (e.g. GitHub). See the Nature Research guidelines for submitting code & software for further information.

Data

Policy information about availability of data

All manuscripts must include a data availability statement. This statement should provide the following information, where applicable:

- Accession codes, unique identifiers, or web links for publicly available datasets
- A list of figures that have associated raw data
- A description of any restrictions on data availability

The data that support the findings of this study are available from the corresponding author upon reasonable request.
HERV peptides affinity to MHC class I molecules was predicted using NetMHCpan version 2.8.
Cancer testis antigen (CTA) library was developed using data available on Ctdatabase (http://www.cta.lncc.br)
Accession numbers for known transcribed HERVs published by Mayer et al. were used to download the reported nucleotide sequences from the National Center for Biotechnology Information's (NCBIs) Entrez database (DB, https://www.ncbi.nlm.nih.gov/Web/Search/entrezfs.html).
Scripts related to data analysis and statistical assessment are available on GitHub https://github.com/SRHgroup/HERV

Field-specific reporting

Please select the one below that is the best fit for your research. If you are not sure, read the appropriate sections before making your selection.

☒ Life sciences ☐ Behavioural & social sciences ☐ Ecological, evolutionary & environmental sciences

For a reference copy of the document with all sections, see nature.com/documents/nr-reporting-summary-flat.pdf

Life sciences study design

All studies must disclose on these points even when the disclosure is negative.

Sample size	The number of patients was chosen based on availability of patient material. For statistical comparison, an equal number of healthy donor samples were included for the analysis.
Data exclusions	No data excluded from analysis.
Replication	All patients and healthy donor samples were screened once with the pMHC library selected according to their HLA type. Virus-specific T cells identified in healthy donors were used as experimental controls. HERV-specific T cells identified in healthy donor was verified using pMHC tetramer staining in an independent experiment.
Randomization	Randomization was not relevant to our study since the patient samples did not derive from a clinical trial.
Blinding	Blinding is not relevant to our study since the patient samples did not derive from a clinical trial.

Reporting for specific materials, systems and methods

We require information from authors about some types of materials, experimental systems and methods used in many studies. Here, indicate whether each material, system or method listed is relevant to your study. If you are not sure if a list item applies to your research, read the appropriate section before selecting a response.

Materials & experimental systems

n/a	Involved in the study	
☐	☒	Antibodies
☒	☐	Eukaryotic cell lines
☒	☐	Palaeontology
☒	☐	Animals and other organisms
☐	☒	Human research participants
☒	☐	Clinical data

Methods

n/a	Involved in the study	
☒	☐	ChIP-seq
☐	☒	Flow cytometry
☒	☐	MRI-based neuroimaging

Antibodies

Antibodies used	CD8-BV480 (BD 566121, clone RPA-T8), dump channel antibodies (CD4-FITC (BD 345768), CD14-FITC (BD 345784), CD19-FITC (BD 345776), CD40-FITC (Serotech MCA1590F), and CD16-FITC (BD 335035)), dead cell marker (LIVE/DEAD Fixable Near-IR; Invitrogen L10119), anti-HLA-A (Nordic BioSite, ACB-5EBF45) , anti-CD107a (BD Bioscience, 555801), IFN-γ- APC (BD Bioscience, 341117) and TNF-α-PE-cy7 (Biolegend, 502930).

Validation	Validation for each antibody was provided by the manufacturer and each antibody was further tested and titrated using human PBMCs to ensure correct performance in the relevant setting. Supplementary figure with gating strategy shows performance of CD8-BV480 and FITC conjugated antibodies, and Figure 4 a-c validates staining of anti-CD107a, IFN-γ- APC and TNF-α-PE-cy7 antibodies.

Human research participants

Policy information about studies involving human research participants

Population characteristics	Peripheral blood was drawn from 22 patients with MDS (n=13), CMML (n=4), and AML (n=5), and from 27 healthy donors. The bone marrow samples were drawn from 12 patients with MDS (n=7) and CMML (n=5) that were enrolled in a clinical study in Australia. All patients were diagnosed according to the World Health Organization (WHO) criteria, and patients with MDS and CMML were risk stratified using the Revised International Prognostic Scoring System (IPSS-R) and CMML-specific prognostic scoring systems (CPSS), respectively.
Recruitment	Patient samples were included for analysis based on availability before and after 5-azacytidine treatment., carrying one or more of the four most common HLA alleles in Caucasian populations (HLA-A*01:01, HLA-A*02:01, HLA-B*07:02, and HLA-B*08:01). All four HLAs were represented evenly. To consider any biases associated with analyzed HLAs, Bayesian analysis were performed to correct for HLA related variations. We don't foresee any other potential biases associated with the samples analyzed in this study.
Ethics oversight	All uses of human material have been approved by the committee on health research ethics in the Capital Region of Denmark and the human research ethics committee of the South Eastern Sydney Local Health District, Australia.

Note that full information on the approval of the study protocol must also be provided in the manuscript.

Flow Cytometry

Plots

Confirm that:

- [x] The axis labels state the marker and fluorochrome used (e.g. CD4-FITC).
- [x] The axis scales are clearly visible. Include numbers along axes only for bottom left plot of group (a 'group' is an analysis of identical markers).
- [x] All plots are contour plots with outliers or pseudocolor plots.
- [x] A numerical value for number of cells or percentage (with statistics) is provided.

Methodology

Sample preparation	Peripheral blood mononuclear cells (PBMCs) were isolated from Peripheral blood immediately after sampling using Ficoll-Paque PLUS (GE Healthcare) density gradient centrifugation and were cryopreserved thereafter. Bone marrow mononuclear cells (BMMCs) obtained from Australian patients were isolated by density gradient centrifugation using Lymphoprep (StemCell Technologies). CD34+ separation was carried out using CD34+ magnetic beads and an AutoMACS Pro Machine (Miltenyi Biotec) according to the manufacturer's instructions.
Instrument	Cells were sorted on a BD FACSAriaFusion or acquired on a BD LSR Fortessa.
Software	FACSDiva software was used to gate and sort the population of interest
Cell population abundance	For every T cell population sorted the sorted cell fraction represented 0.05-30% of the total population
Gating strategy	Lymphocytes were defined within a FSC/SSC plot. Among these we gated on single (FSC-A/FSC-H), live (NIR negative), CD8 positive (BV480) and 'dump' (CD4, 14, 16, 19, and 40) (FITC) negative cells and sorted all mulimer-positive (PE) cells.

- [x] Tick this box to confirm that a figure exemplifying the gating strategy is provided in the Supplementary Information.
