# Supplementary material for: Human endogenous retroviruses form a reservoir of T cell targets in hematological cancers
Source: Nat Commun. 2020 Nov 9;11:5660. doi: 10.1038/s41467-020-19464-8 (PMC7653045; doi:10.1038/s41467-020-19464-8)
Supplement: Supplementary file 4 — Description of Additional Supplementary Files [file 41467_2020_19464_MOESM4_ESM.pdf]

Description of Additional Supplementary Files

Supplementary Data 1. Details of HERVs used in this study to predict T cell antigens.

Supplementary Data 2. Library of HERV-derived peptides.

Supplementary Data 3. CTA peptide library.

Supplementary Data 4. Viral antigen library.

Supplementary Data 5. Details of published RNA-seq data from healthy donor bone marrow samples for HERV expression analysis.
